# Assessment of the Current State of Pharmacovigilance System in Pakistan Using Indicator-Based Assessment Tool

**DOI:** 10.3389/fphar.2021.789103

**Published:** 2022-01-14

**Authors:** Muhammad Akhtar Abbas Khan, Saima Hamid, Tofeeq Ur-Rehman, Zaheer-Ud-Din Babar

**Affiliations:** ^1^ Health Services Academy, Islamabad, Pakistan; ^2^ Fatima Jinnah Women University, Rawalpindi, Pakistan; ^3^ Department of Pharmacy, Quaid-i-Azam University, Islamabad, Pakistan; ^4^ Center for Pharmaceutical Policy and Practice Research, Department of Pharmacy, School of Applied Sciences, University of Huddersfield, Huddersfield, United Kingdom

**Keywords:** pharmacovigilance, system, adverse drug reactions, IPAT, public health, Pakistan, medicine safety, DRAP

## Abstract

**Objectives:** Pakistan felt the need for an effective and robust pharmacovigilance (PV) system after one of the deadliest drug-related tragedies causing more than 300 deaths in 2012. The country set up its national PV center in 2015 and joined WHO’s Program for International Drug Monitoring (PIDM) in 2018 as a full member. The current study was aimed to evaluate the PV system’s functionality, identify the gaps, areas of improvement, and a strategy to lead a functional PV system in Pakistan.

**Methods:** The descriptive cross-sectional study was conducted by providing an interviewer-administered questionnaire of the PV system across Pakistan by utilizing the Indicator based Pharmacovigilance assessment tool (IPAT). By a convenience sampling method 36 study participants were selected from the Drug Regulatory Authority of Pakistan (DRAP), drug administration of provincial health departments of 4 provinces and federally affiliated areas, 5 national public health programs, and 23 public and private hospitals. The assessment includes document review, interviews of the key informants by structured open-ended questions, and a review of websites of relevant organizations.

**Results:** Drug Regulatory Authority of Pakistan (DRAP) with a national PV center received a 75% overall performance score on IPAT. To be regarded as “minimally functioning,” a country’s PV and drug safety system must meet all core indicators. DRAP scored 80.76% on the core indicators so cannot be deemed functional at this time. The only province with a regional PV center, Punjab, had scored 72.13% on relevant parameters. Despite receiving funding from the Global Fund, none of the National Public Health Programs (PHPs) have PV centers or associated activities. All hospitals except two private hospitals could not qualify the minimum requirements for functional PV. The absence of a legal framework for mandatory ADR reporting, lack of drug information center, budgetary constraints, no active surveillance activities, the nonexistence of pharmacovigilance risk assessment expert committee, and insufficient coordination among stakeholders were identified as major gaps.

**Conclusion:** The results of the study reveal that Pakistan’s PV system is not fully functional at all levels. A two-phased strategy encompassing the non-financial and financial interventions is proposed to improve the PV systems at the national, provincial, PHPs, and hospitals levels.

## Introduction

While medicines have benefits, they are also considered to have harmful effects. Though preventable, adverse drug reactions (ADRs) are among the major reasons for death (WHO, 2004). To reduce the risks involved with medicines, pharmacovigilance is considered a key instrument in public health and medical practice (WHO, 2006; 2010). Pharmacovigilance (PV) is a wider discipline and is defined as “*the science and activities relating to the detection, assessment, understanding, and prevention of adverse effects or any other possible drug-related problems*” (WHO, 2002). After the thalidomide catastrophe, there was a global need for speedy transmission of ADR information. As a result of the disaster, several policies, regulations, and amendments, in addition to the WHO Program for International Drug Monitoring (PIDM), were implemented. Several issues emerged in the incident including the hesitant approach of regulatory authorities, poor regulations, and weak review processes. This incident highlighted the significance of a thorough evaluation process and many countries introduced new regulations and strengthened existing drug safety systems and legislation (WHO, 2002; Rice, 2007; Lembit and Santoso, 2010; Beninger and Ibara, 2016).

The scope of pharmacovigilance has been expanding throughout the years from the unrecognized adverse drug reactions to post-market drug surveillance, medication errors, drug quality and therapeutic ineffectiveness (Nwokike and Joshi, 2010), illegal online sale of medicines, unreliable donation of prescription drugs, the growing practice of self-medication, and the sale of counterfeit and fake medicines (WHO, 2002). Pharmacovigilance has evolved as a regulatory activity, through collaboration between the World Health Organization (WHO), the Council for International Organizations of Medical Sciences, and the International Conference on Harmonization (Beninger and Ibara, 2016). Quick approval, prioritization, and expedited review for novel medications have all become more popular in recent years (Darrow et al., 2020). New accelerated and conditional approval routes necessitate more comprehensive and interactive PV, as well as more frequent and creative risk management strategies. FDA is taking extra measures to tackle the new challenges (Pitts, 2015).

Mahmood et al. (2011) underlined the importance of implementing a PV system in Pakistan to reduce drug-related mortality and illness (Mahmood et al., 2011). Until 2012 the country did not have an established PV system. More than 300 people died at the Punjab Institute of Cardiology (PIC) in Lahore in 2011 as a result of tainted medicine Isotab (Isosorbide mononitrate 20 mg). Later, a Judicial Inquiry Tribunal (JIT) was formed to examine the causes of fatalities, and it was discovered that the lack of a PV system and the hospital’s ADR reporting system were the major causes of drug-related adverse events. The Judicial Inquiry Tribunal (JIT) also suggested that PV centers be established at all levels of the health administration department to collect and submit ADRs for rapid risk assessment, appraisal, and management (LHC, 2012).

Pakistan’s PV research is predominantly focused on Knowledge Attitude and Practices (KAP) surveys regarding ADR reporting. Health care professionals (HCPs) have a positive attitude toward medicine safety. However, ADRs are underreported by Pakistani healthcare providers due to poor knowledge of the national ADR reporting system, training, and communication gaps between the hospitals and the regulatory authorities (Iffat et al., 2014; Atif et al., 2016; Hussain et al., 2018; Nisa et al., 2018; Syed et al., 2018). Iftikhar et al. (2018) found a high percentage of Adverse Drug Events (ADEs) among Pakistani adult and pediatric patients with 59.9 and 40.1%, respectively. The study further revealed that most of the ADEs were preventable and associated with medication errors (Iftikhar et al., 2018). Shamim et al. (2016) reported that there were few PV systems at tertiary care level hospitals (Shamim et al., 2016). No study has been conducted on the PV systems of PHPs and health facilities of Pakistan.

**FIGURE 1:**
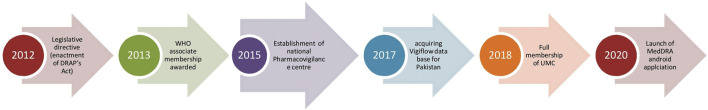
Events of establishment of the national pharmacovigilance center of Pakistan with timelines.

In 2021, Pakistan’s total population is expected to be around 212.48 million (Statista, 2021), with a pharmaceutical industry worth around USD 3.2 billion (The Pakistan Business Council, 2021). More than 600 drug manufacturing licenses (DRAP 2021a) and 80,000 product registrations (DRAP 2021c) have been granted by DRAP. First National PV Center was established in 2015 and DRAP received full membership of Uppsala Monitoring Center (UMC) in 2018 (UMC, 2021a) (Figure 1). The WHO emphasizes the importance of conducting a thorough analysis of the strengths and shortcomings of current PV systems to improve their effectiveness (WHO, 2015). In 2015, Danya found that Pakistan had an ADR collection system in place as well as a PV center (Qato, 2018). Other studies explained that the PV system of Pakistan is at its initial stage of development (Hussain et al., 2018) and needs strengthening and improvement (Shakeel et al., 2014). However, the progress of Pakistan’s current PV system has never been evaluated systematically over time. This study aims to assess Pakistan’s PV system. According to our knowledge, this study is the first of its kind to use the IPAT data collection tool to assess Pakistan’s PV system at the national and provincial levels from its inception through 2020.

## Materials and Methods

### Study Settings

Pakistan has four provinces, i.e., Baluchistan, Khyber Pakhtunkhwa (KPK), Punjab, and Sindh. It has separate health administration for Islamabad Capital Territory (ICT), and two federally affiliated areas, Azad Jammu and Kashmir (AJK) and Gilgit Baltistan (GB). Private and public sectors deliver health services in Pakistan. The health care delivery system is three-tired. The system comprises more than 1200 public sector hospitals, above 5500 basic health units, around 685 rural health centers, and over and above 5800 dispensaries. A large number of private hospitals and stand-alone clinics operate separately. The workforce comprises 195,896 doctors, more than 95,000 lady health workers, 99,228 nurses, and 34,000 pharmacists (Muhammad et al., 2021; WHO, 2021a).

### Study Design and Sampling

We conducted structured interviews of key informants of PV for our descriptive cross sectionals study across Pakistan during July-December 2020. By convenience sampling method 36 study participants were selected from DRAP, drug administration of provincial health departments, ICT, AJK, and GB, Public Health Programs (PHPs), and public and private hospitals (see Supplementary Table). The majority of respondents were pharmacists working in federal and provincial drug administrations, the chief pharmacists working in hospitals, logistic support managers, and program managers in PHPs.

PV activities at the DRAP and five PHPs, including the National Malaria Control Program (NMCP), the National Aids Control Program (NACP), the National Tuberculosis Control Program, the Expanded Program on Immunization (EPI), and the Pakistan Polio Eradication Initiative (PPEI) were evaluated at the national level, while each administrative unit of Pakistan, including AJK, Baluchistan, GB, ICT, KPK, Punjab, and Sindh, was evaluated at the provincial level.

IPAT suggests sampling of 10–15 health facilities, to collect representative data on PV activities at all levels of health delivery. A total of 23 health facilities, including 8 private and 15 public or government hospitals, were selected. Private hospitals include Agha Khan University Hospital (AKUH) Karachi, Quaid-e-Azam International Hospital (QIH), and Shifa International Hospital (SIH) in Islamabad, Shaukat Khanum Memorial Cancer Hospital (SKMCH) in Lahore, Rehman Medical Institute (RMI) Peshawar, Agha Khan Medical Center (AKMCG) Gilgit, Baluchistan Institute of Nephrology and Kidney Transplant (BINIQ) Quetta, and Riaz Hospital (RHM) Mirpur, AJK. While government hospitals include Allied Hospital (AH) Faisalabad, Benazir Bhutto Shaheed Hospital (BBH), District Headquarter Hospital (DHH) and Holy Family Hospital (HFH) in Rawalpindi, Federal Government Polyclinic Hospital (FGPH), and Pakistan Institute of Medical Sciences (PIMS) in Islamabad, Children Hospital (CH), Jinnah Hospital (JH), and Punjab Institute of Cardiology (PIC) in Lahore, Jinnah Postgraduate Medical Centre (JPMC) and National Institute of Child Health (NICH) in Karachi, Hayatabad Medical Complex (HMC) Peshawar, DHQ Hospital (DHQHG) Gilgit, Bolan Medical Complex Hospital (BMCH) Quetta, and DHQ Teaching Hospital (DHQTH) Mirpur AJK. This research is carried out without patients, carers, or members of the public.

### Data Collection

#### Data Collection Tool

Data was collected using the IPAT developed and validated by “management sciences for health (MSH)” under a USAID program to examine PV systems in developing countries. IPAT consists of a total of 43 indicators with 26 core and 17 supplementary indicators. These indicators focus on five areas of the PV system, i.e., (1) policy, law, and regulation (four indicators); (2) systems, structures, and stakeholder coordination (15 indicators); (3) signal generation and data management (six indicators); (4) risk assessment and evaluation (eight indicators); and (5) risk management and communication (10 indicators). The indicators are further categorized by “structure,” “process,” and “outcome.” The tool’s objective is to make PV assessment easier by asking questions about the PV system (SPS Program, 2009).

The first section (“policy, law, and regulation”) is intended to assess the National Regulatory Authority as DRAP. As a result, only the four other sections are relevant to provincial drug administration, PHPs, and health facilities. For our study, we selected the following relevant indicators according to the study settings.

• 42 indicators for DRAP (1.1–1.4, 2.1–2.11, 2.13–2.15, 3.1–3.6, 4.1–4.8, 5.1–5.10)
• 37 indicators for Provincial Health Department (2.1–2.11, 2.13–2.14, 3.1–3.6, 4.1–4.8, 5.1–5.10)
• 30 indicators for health facilities
• 31 indicators for Public Health Programs

#### Data Collection Process

We approached participants directly and over the phone before data collection to ask them if they would participate in the study. Participants were interviewed in-person to provide information on the indicators that were featured on the IPAT tool they used. There were also open-ended questions about the current PV system in the questionnaires, apart from the indicators-related items. As evidence supporting the interviews, PV-related documents were obtained from the participants. Additional information was gathered from the websites of participating organizations and reviews of documents such as the Drugs Act 1976, the DRAP Act 2012, Pakistan National PV guidelines, the draft PV Rules 2020, DRAP’s Newsletter, Punjab PV plans 2017 and 2019, the fundamentals of PV and its emergence in Punjab, Punjab drug information bulletins, Guidelines of PHP, and National Health Vision Pakistan 2016–2025.

#### Data Analysis

Microsoft Excel was used to calculate the results, following quantitative and qualitative analyses of the data. Each IPAT data collection tool indicator has a number and percentage with suggested criteria. The responses of participants are recorded as either a “Yes” or a “No.” Any fulfilled core indicator is given 2 points, supplementary indicator 1 point, and any unfulfilled indicator is given 0 points. The maximum points for core and supplementary indicators are 52 and 17, respectively. These numerical values have been assigned according to IPAT tool scoring. The threshold for various quantitative indicators (2.13, 4.4, 5.3, 5.4, 5.5, 5.6, 5.7, and 5.10) was not set due to the small values of the data. Finally, the response data is tabulated and also displayed as a column-chart and radar chart to allow for visual identification of progress over time. The value was multiplied by 100 after the final score was calculated by combining the scores of all indicators and dividing it by the aggregate score of all indicators.

## Results

### The Drug Regulatory Authority of Pakistan

**FIGURE 2:**
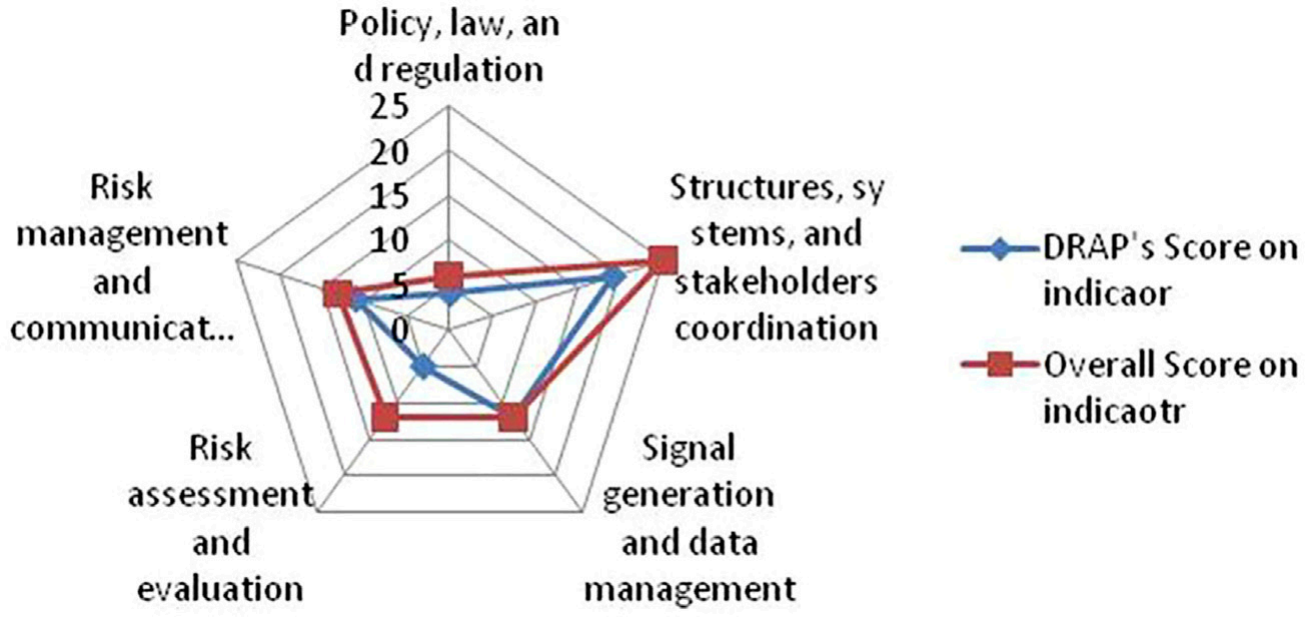
Five-axis spider-diagram (0–25 score) showing the DRAP’s scores on five main indicators of IPAT.

The Drug Regulatory Authority of Pakistan (DRAP) was evaluated for 42 PV indicators which contain 26 core and 16 supplementary indicators, for a total score of 68. DRAP achieved an aggregate score of 75% (Table 1) with breakup into four categories: (1) “policy, law, and regulation” (66.6%), (2) “Structures, systems, and stakeholder’s coordination” (76%), (3) “Signal generation and data management” (100%), (4) “Risk assessment and evaluation” (41.66%), and (5) “Risk management and communication” (84.61%), (Figure 2). Overall, the DRAP met (21/26) 80.76% of the core and (9/16) 56% of supplementary indicators.

**TABLE 1:** Drug regulatory authority of Pakistan.

Pharmacovigilance indicators at the national level		Core/supplementary	Score
1	Policy, law, and regulation		
1.1	Existence of a national policy document addressing pharmacovigilance	C	2
1.2	Specific pharmacovigilance provisions in national medicines or similar laws	C	2
1.3	Legal requirements require marketing licensors to report all serious adverse reactions to the national drug regulator	S	0
1.4	Legal requirement for the marketing authorization holder to conduct post-marketing surveillance activities	S	0
Subtotal score (%)			4/6 (66.6)
2	Structures, systems, and stakeholders coordination		
2.1	Pharmacovigilance center exists	C	2
2.2	Clear mandate, structure, roles, and responsibilities of pharmacovigilance center exists	C	2
2.3	Medicine information service exists	C	0
2.4	Separate staff for pharmacovigilance	C	2
2.5	A dedicated budget for pharmacovigilance exists	C	0
2.6	National medicine safety advisory committee exists	C	0
2.7	National pharmacovigilance guidelines exists	C	2
2.8	SOPs for safe use of medicines exists	C	2
2.9	Basic communication tools provided for reporting and information on the safety of medicines	C	2
2.10	Drug safety bulletin exists	C	2
2.11	Reference materials available in pharmacovigilance center	S	1
2.13	Training of healthcare professionals on pharmacovigilance during the previous year	S	1
2.14	Countrywide platform or plan for coordinating pharmacovigilance initiatives	C	2
2.15	Membership of national pharmacovigilance center of WHO International Drug Monitoring program	S	1
Subtotal score (%)			19/25 (76)
3	Signal generation and data management		
3.1	A mechanism for coordinating and compiling pharmacovigilance data from all sources across the country	C	2
3.2	Database for tracking pharmacovigilance activities exists	C	2
3.3	A form for reporting suspected ADRs exists	C	2
3.4	A form for reporting suspected product quality issues exists	C	2
3.5	A form for reporting suspected medication errors exists	C	2
3.6	A form for reporting suspected treatment failure exists	C	2
Subtotal score (%)			12/12 (100)
4	Risk assessment and evaluation		
4.1	Last year, a medicine utilization review performed	S	0
4.2	Within the previous 5 years, a survey for pharmaceutical product quality undertaken	S	1
4.3	Medication errors quantified in the last year	S	0
4.4	Number of ADR reports collected in the last year	C	2
4.5	Active surveillance activities conducted during the last 5 years	C	0
4.6	Public health programs reported ADEs for patients in the last year	C	2
4.7	Public health programs modified the treatment of patients due to ADRs in the last year	C	0
4.8	Public health programs reported serious ADEs of patients in the last year	S	0
Subtotal score (%)			5/12 (41.66)
5	Risk management and communication		
5.1	Risk mitigation plans targeted at high-risk medicines	S	0
5.2	Prequalification schemes for procurement of medicines	S	1
5.3	In the last year, medicine safety information requests received and addressed	S	1
5.4	Medicine safety bulletin published in the last year	S	1
5.5	Medicine safety issues addressed on external information	S	1
5.6	Safety alerts including “Dear healthcare professional” developed and distributed in the last year	S	1
5.7	The average time lag between identification of safety signal of a serious ADR or significant medicine safety issue and communication to healthcare workers and the public	C	2
5.8	Percentage of Drug and Therapeutics Committees that handled medicine safety issues during last year	C	2
5.9	Last year’s public or community education initiatives on medication safety	S	0
5.10	Medicines sampled in the last year that passed product quality tests	C	2
Subtotal score (%)			11/13 (84.61)
Total			51/68 (75%)

According to the study findings, DRAP has established a national PV center with seven designated staff members, standard operating procedures, and guidelines. With full membership of the WHO PIDM in 2018, the collected data is transferred to the VigiBase. In addition to the DRAP MED Vigilance E-Reporting System and the Web-RADR Med Safety mobile application, the National PV Center has provided online and manual ADR reporting forms. Safety alerts and advisories are issued on DRAP’s website and social media accounts. During 2016–2018 DRAP has taken several regulatory actions on external safety information (Table 2). Since its establishment, 6587 ADR reports (116 in 2018, 2415 in 2019, and 4056 in 2020) have been received by the NPC. The majority of reports are from pharmaceutical firms, with 124 ADRs coming from Punjab’s PV center. Not a single ADR report from the public has been reported.

**TABLE 2:** Regulatory actions by DRAP.

S. No.	Drug	Basis for action	Source of information	Action taken
1.	Systemic fluoroquinolones (DRAP, 2016)	Risk of disabling adverse effects of tendons, muscles, joints, and central nervous system	US-FDA FDA	Prescribing information and labeling information updated, black box warning
2.	Hydroxyzine hydrochloride (DRAP, 2017b)	Abnormal cardiac rhythm	European Medicine Agency (EMA), United Kingdom (MHRA), PMDA (Japan), Health Canada	Prescribing information updated and daily dose reduced
3.	Direct acting hepatitis cantiviral (DRAP, 2017a)	Risk of hepatitis b virus (hbv) reactivation	PMDA (Japan) US-FDA	Prescribing information updated and box warning
4.	Irrational combination of Paracetamol 500 mg, Thioridazine 3 mg, and caffeine 70 mg (DRAP, 2016)	Withdrawn of Thioridazine worldwide by the brand leader Novartis. Combination not registered in any Stringent Regulatory Authority	Internal review and Novartis Pharma	Cancellation of Registration
5.	Oral ketoconazole (DRAP, 2016)	Potential to cause severe liver injuries	USFDA, European Medicines Agency’s Committee (EMA), Health Canada	Cancellation of Registration
6.	Clarithromycin (DRAP, 2018)	A possible increase in the risk of heart disease	US-FDA	Prescribing information updated
7.	Canagliflozin (DRAP, 2018)	Risk of amputation of lower limb	US-FDA. European Medicines Agency (EMA)	Prescribing information updated, black box warning

DRAP lacks legal provisions requiring medicine registration holders to report ADRs to the DRAP, a medicine information center, and a dedicated budget for PV-related activities. Pharmacovigilance Risk Assessment Expert Committee is also missing, however, an internal “Causality Assessment and Signal Review group” comprised of DRAP officers has been notified.

### Pharmacovigilance Activities at Provincial Health Departments

**FIGURE 3:**
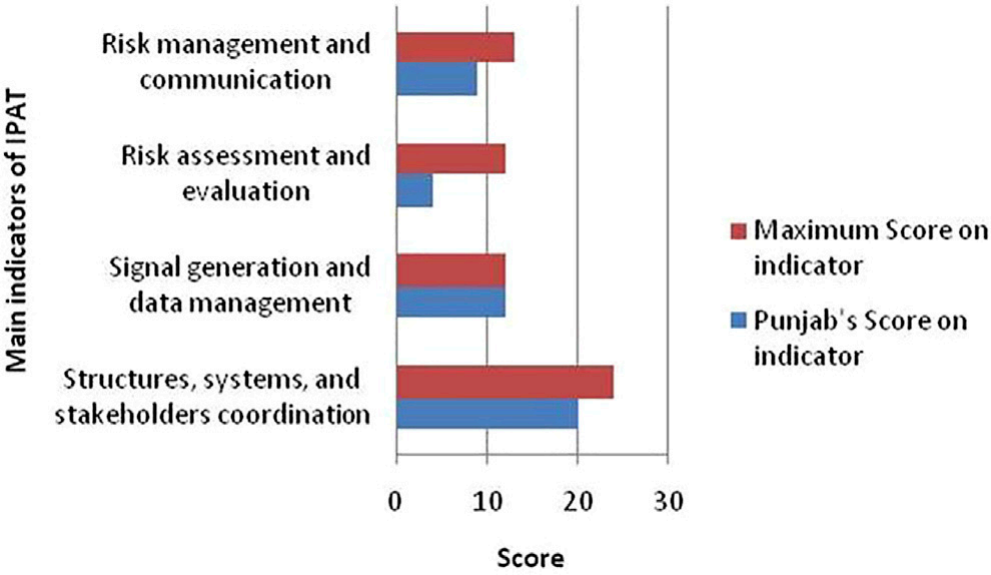
Score of Punjab PV center on main indicators of IPAT.

It was found that except Primary & Secondary Healthcare Department (PSHD) Punjab and Health Department of Islamabad Capital Territory (ICT) all health departments of other administrative units of Pakistan have no PV center and therefore no PV-related activities are carried out. The PSHD Punjab was assessed on 37 PV indicators, with 24 core and 13 supplemental indicators aggregating 61 points. Punjab scored (45/61) 72.13% overall (Table 3), with the following categories: (1) “structures, systems, and stakeholder coordination” (83.33%), (2) “signal generation and data management” (100%), (3) “risk assessment and evaluation” (25%), and (4) “risk management and communication” (69.23%) (Figure 3). Overall, Punjab satisfied 79.16% of the core indicators (19/24) and 53.84% of the supplemental indicators (7/13) in total.

**TABLE 3:** Primary and secondary healthcare department Punjab.

Pharmacovigilance indicators at the provincial level		Core/supplementary	Score
2	Structures, systems, and stakeholders coordination		
2.1	Pharmacovigilance center exists	C	2
2.2	Clear mandate, structure, roles, and responsibilities of pharmacovigilance center exists	C	2
2.3	Medicine information service exists	C	0
2.4	Separate staff for pharmacovigilance	C	2
2.5	A dedicated budget for pharmacovigilance exists	C	0
2.6	National medicine safety advisory committee exists	C	2
2.7	National pharmacovigilance guidelines exists	C	2
2.8	SOPs for safe use of medicines exists	C	2
2.9	Basic communication tools provided for reporting and information on the safety of medicines	C	2
2.10	Drug safety bulletin exists	C	2
2.11	Reference materials available in pharmacovigilance center	S	1
2.13	Training of healthcare professionals on pharmacovigilance during the previous year	S	1
2.14	Countrywide platform or plan for coordinating pharmacovigilance initiatives	C	2
Subtotal score (%)			20/24 (83.33)
3	Signal generation and data management		
3.1	A mechanism for coordinating and compiling pharmacovigilance data from all sources across the country	C	2
3.2	Database for tracking pharmacovigilance activities exists	C	2
3.3	A form for reporting suspected ADRs exists	C	2
3.4	A form for reporting suspected product quality issues exists	C	2
3.5	A form for reporting suspected medication errors exists	C	2
3.6	A form for reporting suspected treatment failure exists	C	2
Subtotal score (%)			12/12 (100)
4	Risk assessment and evaluation		
4.1	Last year, a medicine utilization review performed	S	0
4.2	Within the previous 5 years, a survey for pharmaceutical product quality undertaken	S	1
4.3	Medication errors quantified in the last year	S	0
4.4	Number of ADR reports collected in the last year	C	2
4.5	Active surveillance activities conducted during the last 5 years	C	0
4.6	Public health programs reported ADEs for patients in the last year	C	0
4.7	Public health programs modified the treatment of patients due to ADRs in the last year	C	0
4.8	Public health programs reported serious ADEs of patients in the last year	S	0
Subtotal score (%)			3/12 (25%)
5	Risk management and communication		
5.1	Medicine safety bulletin published in the last year	S	0
5.2	Medicine safety issues addressed on external information	S	0
5.3	Safety alerts including “Dear healthcare professional” developed and distributed in the last year	S	1
5.4	The average time lag between identification of safety signal of a serious ADR or significant medicine safety issue and communication to healthcare workers and the public	S	1
5.5	Percentage of Drug and Therapeutics Committees that handled medicine safety issues during last year	S	1
5.6	Last year’s public or community education initiatives on medication safety	S	1
5.7	Medicine safety bulletin published in the last year	C	2
5.8	Medicine safety issues addressed on external information	C	2
5.9	Safety alerts including “Dear healthcare professional” developed and distributed in the last year	S	0
5.10	Medicines sampled in the last year that passed product quality tests	C	2
Subtotal score (%)			9/13 (69.23)
Total			45/61 (73.77%)

The provincial drug control unit of PSHD Punjab has established a provincial pharmacovigilance center (PPC) along with designated five officers, PV guidelines, and SOPs. A monthly Punjab drug safety newsletter is published regularly. Punjab also has constituted an ADR risk management and scrutiny committee to scrutinize the Individual Case Safety Reports (ICSRs). Drug Safety Alerts are posted on a Drug Control Unit’s webpage. Punjab also uses Facebook and Twitter to disseminate safety information. Furthermore, KPK is in the process of developing an ADR collection system, while Baluchistan has yet nominated focal persons at the provincial and district levels. In 2018, ICT drug administration set up a PV center and signed MOU with 11 private hospitals of Islamabad for ADR reporting. Nearly 23 focal persons from the hospitals were trained on PV-related activities; despite all these efforts, the PV Center of ICT has not received any ADR.

### Pharmacovigilance Activities at Public Health Programs

The quantitative results have not been computed because only a few indicators of assessment tools were verified in each program. That is why the findings are not summarized in a table or shown as a chart. The key informants were interviewed with a structured IPAT questionnaire and for additional information, the program manager and procurement officer/logistic support officer were interviewed through unstructured questions, and results are presented through the qualitative description. All vertical programs except EPI and Initiative for the eradication of polio have pharmacists in their staff.

### National Malaria Control Program

The Directorate of NMCP has no PV unit and designated staff responsible for data management. No form to report ADR, problems with product quality, medication error, and treatment failure was available. The procurement of medicines is based on WHO prequalification criteria due to global fund requirements. The strategic plan for malarial control in Pakistan (2015–2020) does not account for ADRs reporting or medicine safety. Two separate studies were conducted including an assessment of therapeutic efficacy and safety of an anti-malarial drug (Directorate of malaria control Pakistan, 2017) and the quality of anti-malarial drugs. In one of the survey-based studies, the clinical safety of an anti-malarial drug was assessed.

### National Tuberculosis Control Program

NTBCP also lacks a PV center; however, the procurement officer who is a pharmacist is assigned the additional responsibility of monitoring medicine-related issues. There is a form available for reporting suspected treatment failure (TB-07) and ADRs; however, the separate subset of other forms is not available for product quality-related problems and medication errors. The data for the number of ADR reports during last year was not available. Only treatment failure information was collected which was 3% last year. Only one medicine, i.e., vitamin B-6 was withdrawn from the market in 2018 due to quality-related issues.

### National AIDS Control Program

The pharmacovigilance center and designated staff are not provided in the NACP. Quality assurance guidelines contain the statement regarding ADRs reporting. It is the responsibility of the antiretroviral therapy physicians to report any ADR. An internal form is available for reporting ADRs, product-related quality issues, medication errors, and suspected treatment failure. Less than 1% of patients had treatment failure during the last year. The procurement is mandated through WHO qualification.

### Pakistan Polio Eradication Initiative

The PV center is not physically present. There is no ADR reporting form, however, through the online “contact us” form, anyone can submit a query related to the polio vaccine and its suspected effects.

### Expanded Program on Immunization

There is no formal PV center, however, a monitoring and evaluation (M&E) wing is responsible for AEFIs. The WHO’s SOPs for vaccine safety are being used. A quarterly bulletin is published since August 2017 and provinces issue their bulletins. During the last measles vaccination campaign the vaccinators were trained at the Union Council level for reporting AEFIs. There are case reporting forms and case investigation forms. A total of 2272 cases of AEFIs were reported in 2018. Most of the ADRs were coincidental. The WHO prequalification is not mandatory for procurements. An AEFI review committee is established at the national level.

### Pharmacovigilance Activities at Health Facilities (Hospitals)

PV activities were assessed at 23 different health facilities that were selected at random. A hospital is considered a minimally functional health facility if it achieves a score of 38 on the IPAT data collection tool for health facilities, which consists of 30 PV indicators, 19 core, and 11 supplementary indicators. Since one private and two public hospitals [Agha Khan University Hospital (AKUH), Jinnah Hospital (JH) Lahore, and Children Hospital (CH) Lahore, respectively] did not respond to the IPAT data collection tool, the response rate of health facilities was observed to be 87%.

In contrast to the majority of private-sector hospitals, four private [i.e., Quaid-e-Azam International Hospital (QIH), Agha Khan Medical Center (AKMCG) Gilgit, Baluchistan Institute of Nephrology and Kidney Transplant (BINIQ) Quetta, and Riaz Hospital (RHM) Mirpur, AJK] and nine public hospitals [i.e., Benazir Bhutto Shaheed Hospital (BBH), District Headquarter Hospital (DHH), and Holy Family Hospital (HFH), Federal Government Polyclinic Hospital (FGPH), and Pakistan Institute of Medical Sciences (PIMS) in Islamabad, Punjab Institute of Cardiology (PIC) Lahore, DHQ Hospital (DHQHG) Gilgit, Bolan Medical Complex Hospital (BMCH) Quetta, and DHQ Teaching Hospital (DHQTH) Mirpur AJK] at both the federal and provincial levels were found to be lacking in PV centers and related activities, so no response was tabulated or displayed in the radar chart. However, three private [i.e., Rehman Medical Institute (RMI), Shifa International Hospital (SIH), and Shaukat Khanum Memorial Cancer Hospital (SKMCH)] and four public [i.e., Allied Hospital (AH), Hayatabad Medical Complex (HMC), Jinnah Postgraduate Medical Centre (JPMC), and National Institute of Child Health (NICH)] hospitals responded to the IPAT data collection tool, and their responses are tabulated in Table 4. PV activities of healthcare facilities decrease in the following order: Hospital [score (%)]: SKMCH [41 (83.6)] > SIH [38 (77.5)] > AH [29 (59)] > NICH [23 (46.9)] > HMC and RMI [14 (28.5)]. SKMCH received the highest scores, SIH is a minimally functional health facility, while AH, and HMC, NICH, and RMI received lower scores than a minimally functional health facility. Private hospitals that do have PV systems do not share ADR-related data with national or provincial PV centers.

**TABLE 4:** Health facilities pharmacovigilance indicators.

30 Pharmacovigilance indicators			Health facilities							Total*	Achieving indicator (% HF)**
			A	B	C	D	E	F	G		
1	(2.1)	Pharmacovigilance center exists	2	2	2	2	2	0	0	1.42	71
2	(2.2)	Clear mandate, structure, roles, and responsibilities of Pharmacovigilance center exists	2	2	0	0	2	0	0	0.85	42.5
3	(2.3)	Medicine information service exists	2	2	0	2	2	2	2	1.71	85.5
4	(2.4)	Separate staff for pharmacovigilance	2	2	2	2	2	0	0	1.42	71
5	(2.5)	A dedicated budget for pharmacovigilance exists	0	0	0	0	0	0	0	0	0
6	(2.8)	SOPs for safe use of medicines exists	2	2	0	0	2	2	2	1.42	71
7	(2.9)	Basic communication tools provided for reporting and information on the safety of medicines	2	2	2	2	2	0	0	1.42	71
8	(2.10)	Drug safety bulletin exists	0	2	0	0	2	0	0	0.57	28.5
9	(2.11)	Reference materials available in pharmacovigilance center	1	1	1	1	1	1	1	1	100
10	(2.13)	Training of healthcare professionals on pharmacovigilance during the previous year	1	1	0	0	1	0	0	0.42	42
11	(3.3)	A form for reporting suspected ADRs exists	2	2	2	2	2	2	2	2	100
12	(3.4)	A form for reporting suspected product quality issues exists	0	0	0	0	2	2	2	0.85	42.5
13	(3.5)	A form for reporting suspected medication errors exists	2	2	0	0	2	2	2	1.42	71
14	(3.6)	A form for reporting suspected treatment failure exists	0	0	0	0	2	0	0	0.28	14
15	(4.1)	Last year, a medicine utilization review performed	0	1	0	1	1	0	0	0.42	42
16	(4.3)	Medication errors quantified in the last year	0	1	1	1	1	0	0	0.57	57
17	(4.4)	Number of ADR reports collected in the last year	0	2	0	0	2	0	0	0.57	28.5
18	(4.5)	Active surveillance activities conducted during the last 5 years	0	2	0	2	0	0	0	0.57	28.5
19	(4.6)	Public health programs reported ADEs for patients in the last year	2	0	0	0	2	0	0	0.57	28.5
20	(4.7)	Public health programs modified the treatment of patients due to ADRs in the last year	2	2	0	0	2	0	0	0.85	42.5
21	(4.8)	Public health programs reported serious ADEs of patients in the last year	1	1	1	0	1	0	0	0.57	57
22	(5.1)	Risk mitigation plans targeted at high-risk medicines	1	1	1	1	1	1	1	1	100
23	(5.3)	In the last year, medicine safety information requests received and addressed	1	0	0	1	1	0	0	0.42	42
24	(5.4)	Medicine safety bulletin published in the last year	0	1	0	0	1	0	0	0.28	28
25	(5.5)	Medicine safety issues addressed on external information	0	1	0	0	1	0	0	0.28	28
26	(5.6)	Safety alerts including “Dear healthcare professional” developed and distributed in the last year	0	1	0	0	1	0	0	0.28	28
27	(5.7)	The average time lag between identification of safety signal of a serious ADR or significant medicine safety issue and communication to healthcare workers and the public	2	2	2	2	2	2	2	2	100
28	(5.8)	Percentage of Drug and Therapeutics Committees that handled medicine safety issues during last year	2	2	0	2	0	0	0	0.85	42.5
29	(5.9)	Last year’s public or community education initiatives on medication safety	0	1	0	0	1	0	0	0.28	28
30	(5.10)	Medicines sampled in the last year that passed product quality tests	0	0	0	2	0	0	0	0.28	14.8
Total score for minimally functional health facility (38)/Total maximum score (49)			29	38	14	23	41	14	14	—	—

A, Allied Hospital, Faisalabad; B, Shifa International Hospital, Islamabad; C, Jinnah Postgraduate Medical Centre, Karachi; D, National Institute of Child Health, Karachi; E, Shaukat Khanum Memorial Cancer Hospital, Lahore; F, Hayatabad Medical Complex, Peshawar; G, Rehman Medical Institute, Peshawar; 0, absence of core/supplementary indicator; 1, presence of supplementary indicator; 2, the presence of core indicator. *Total = Average sum/7; **Percent health facility achieving indicator = 100 x total/a (a = 1 or 2 for supplementary or core indicator, respectively).

## Discussion

The study examines Pakistan’s present PV system. Although no performance criterion has been set to define an effective PV system in a specific country, however, IPAT recommends that the PV system of a country must meet all of the core indicators to be considered minimally functional (SPS Program, 2009). DRAP attained (21/26) 80.76% score of the core indicators and, therefore, at the moment cannot be considered functional. Similar results are reported during the evaluation of PV systems for two African countries (Kabore et al., 2013; Constant Allabi and Nwokike, 2014) and Nepal by using the IPAT (Jha et al., 2021). However, in comparison to the survey conducted by Danya (2015) DRAP has improved few indicators of risk assessment and evaluation (Qato, 2018).

The NPC currently employs seven officers. This figure is insufficient in comparison to the number of registered medications in the country and the growing number of ADR reports. A study found less than 10 trained personnel in more than 80% of study participant countries (Olsson et al., 2010). The scarcity of trained staff affects the data management and performance of PV systems (Wilbur, 2013) and is the reason for unsuccessful experiences (Chejor, 2018).

The DRAP’s NPC has received 6587 ADR reports in total. However, this number is much lower than required as per WHO standards which indicate that over 200 reports per one million inhabitants are produced annually by the countries with the best reporting rates. Considering more than 200 million people, the size of Pakistan’s population, a total of 40,000 reports annually are predicted (Syed et al., 2018). Not a single ADR is received from the general population, however, a limited number of ADRs were reported by HCPs, which indicates a need for their PV education, awareness, and training. DRAP has provided an e-reporting system and Android application for ADR reporting, and it is obligatory to explore the reasons and barriers for non-reporting by the general public. An annual increase in the number of ADR reports can be seen from 2018 to 2020. The number of reports received in 2019 is 20 times higher than in 2018, and nearly two times higher in 2020 compared with 2019. It may be implied that as Pakistan’s PV system advances, the number of ADR reports will increase. The WHO’s threshold of ADR reports per million can be achieved in Pakistan over a certain period of time provided that a sustainable PV system is implemented. Similarly, another study reveals that long-term tradition of ADR reporting increases the number of reports (Glamočlija et al., 2018). The low number of ADR reports in Pakistan is due to various factors including non-reporting by the physicians. Hussain et al. (2021) informed that physicians and nurses of a teaching hospital did not report any ADRs during 1 year (Hussain et al., 2021). Other studies show similar results (Bäckström et al., 2000; Oshikoya and Awobusuyi, 2009). In various studies conducted in Pakistan, physicians, pharmacists, and nurses all cited fear of legal consequences as a major barrier to reporting (Khan et al., 2015; Nisa et al., 2018; Hussain et al., 2018).

Both DRAP and the directorate of drug control, PSHD Punjab issue newsletters, communicate safety alerts on their websites (DRAP 2021b; 2019; Provincial Quality Control Unit Punjab, 2021) and social media accounts like Facebook and Twitter which can help in improving the ADR reporting as several studies suggest that designated social media websites/apps like Twitter and WhatsApp connected with national and regional PV centers can help in collecting ADRs (Daley et al., 2018; Shrestha et al., 2019; Hussain, 2021; Meher, 2021).

Measurement in PV has shifted from the traditional measurement of operational performance to measuring the specific regulatory action. The stringent regulatory authorities are focusing on risk minimization measures. The ultimate test for the PV system is a demonstration of public health benefits. DRAP always takes into account the safety reports coming from outside sources for regulatory actions (Table 2). It shows DRAP pledges to patient safety and access to quality medicines.

A country’s commitment to medicines’ safety can be gauged with the existence of PV policy. Similarly, the enactment of regulations ensures the legal cover to the monitoring and compliance by all stakeholders (Nwokike and Eghan, 2010). There is a clear policy of DRAP on medicine safety. However, the legislation requiring mandatory ADR reporting by stakeholders is missing. The ADR reporting system in Pakistan is voluntary. At the moment DRAP cannot enforce mandatory reporting by all stakeholders until the federal government of Pakistan approves the draft pharmacovigilance rules.

To provide independent scientific advice and guidance on medication safety a functional national advisory committee is required. The Pharmacovigilance Risk Assessment Expert Committee (PRAEC) is not established since the establishment of NPC in 2015. However, a “Causality Assessment and Signal Review group” comprised of DRAP officers defining the internal working has been notified. The absence of PRAEC is one of the major reasons that no safety signal has been generated on ICSRs by Pakistan (UMC, 2021b).

The WHO recommends that a better way of collecting spontaneous ADR reports is by using regional PV centers. Communication regarding medicine safety also works well at regional centers with short communication lines with HCPs (UMC, 2000). Pharmacovigilance centers are also absent from all provincial health departments except for Punjab. Only Punjab is sharing medicines safety reports with DRAP where half of the country’s population lives. PV activities are believed to be solely the responsibility of the DRAP by provincial health administrative units; this perception needs to be altered, potentially through education, training, and active interactions.

Even though most Pakistan’s national health programs are funded by the global fund (The Global Fund, 2020), the formal PV system is lacking in all programs. According to the WHO, PV should be a part of every PHP, to optimize the usage of limited health resources and avoid possible medicine-related catastrophe (WHO, 2006). One of the reasons for the absence of PV activities at PHPs is that PV is not duly included in the funding proposal as seen in the strategic plan for the Malaria Control Program Pakistan, 2015–2020 (Directorate of malaria control Pakistan, 2014), which lacks any provision for reporting ADRs. Likewise, Stergachis et al. (2010) assessed proposals and operational plans of 15 countries for global fund malaria and the US president’s malaria initiative and found that PV-related activities and financial support requests are not included adequately and consistently (Stergachis et al., 2010).

Allegations of vaccine-related adverse events that are not promptly and thoroughly addressed can erode vaccination faith and have far-reaching implications for immunization coverage and disease incidence (WHO, 2021b). Pakistan is one of the two countries along with Afghanistan that is struggling to get polio-free. Conspiracy rumors about the polio vaccine ADRs also play a pivotal role in the public for not trusting the vaccine. In a controversy that emerged in 2019 in Peshawar, Pakistan, hundreds of children rushed to hospitals with abdominal problems and fainting following the immunization. The angry protesters torched a health center. The government responded immediately and a key conspirator was arrested for his involvement in spreading the rumors. To gain the trust of parents on immunization it was proposed to disseminate information on the number of administered vaccine doses and their ADRs (Ali et al., 2019). This incident highlighted the importance of risk management and communication.

To our knowledge, no research has ever been undertaken to evaluate the PV systems of health facilities in Pakistan. The majority of the studies are conducted to measure the knowledge attitude and practices of HCPs (Iffat et al., 2014; Raza and Jamal, 2015; Atif et al., 2016; Hussain et al., 2018; Nisa et al., 2018; Syed et al., 2018; Muhammad et al., 2021) PV systems are found missing in almost all of Pakistan’s public sector hospitals. This may be because of the non-availability of ADR forms in hospitals (Nisa et al., 2018; Syed et al., 2018), poor knowledge and ADRs reporting practices of health care professionals (Nisa et al., 2018), lack of knowledge about ADR reporting systems in the country, the gap between hospitals and regulators in terms of training and communication (Hussain et al., 2018), and concerns over legal responsibility (Mustafa et al., 2013). The irony is that the Punjab Institute of Cardiology where the Isotab tragedy took place has no PV system or activities. Our results show that not all hospitals have a budget set aside for PV-related activities. Similar findings are explained in a study conducted in the south-south zone of Nigeria (Opadeyi et al., 2018).

It was observed that DRAP responded to the Isotab (isosorbide mononitrate 20 mg) (LHC, 2012) and Tyno cough syrup (chlorpheniramine maleate and dextromethorphan 15 mg/5 ml) (Tribune, 2012) events but not in the same way that the rest of the world has to the thalidomide tragedy. We proposed two stages of framework for the advancement of the PV system in Pakistan. The approval of draft PV Rules and the establishment of a Pharmacovigilance Risk Assessment Expert Committee require no financial investment at the first level. Step two involves initiatives such as the development and strengthening of PV centers at national, provincial, PHPs, and hospitals levels, the recruitment of trained staff, planned PV training programs, and developing alliances with universities to perform a drug utilization review or active surveillance activities, all of which entail financial investment.

There are some limitations of the study. To verify the information, the respondent’s replies are considered unless verified from the documentary evidence. IPAT carries some inherent limitations related to the non-establishment of sensitivity and specificity of indicators. Due to convenience sampling, the data may not represent the whole country. Despite all these facts, the study provides a basis for further research to explore challenges and barriers in the approval of PV regulation through in-depth interviews.

In conclusion, the study revealed that the pharmacovigilance system of Pakistan is not meeting the minimum standards. Public health programs and health facilities need to set up PV systems and integrate them with the national PV center.

## Data Availability

The original contributions presented in the study are included in the article/Supplementary Material. Further inquiries can be directed to the corresponding author.

## References

[B1] Ali M. Ahmad N. Khan H. Ali S. Akbar F. Hussain Z. (2019). Polio Vaccination Controversy in Pakistan. Lancet 394, 915–916. 10.1016/S0140-6736(19)32101-4 31526731

[B2] Atif M. Arslan B. Quratulain S. Zainab A. Aminah N. Sonia A. (2016). Knowledge, Attitude, and Practices of Health Care Professionals Regarding Pharmacovigilance in Pakistan. Value Heal. 19, A278. 10.1016/j.jval.2016.03.1979

[B3] Bäckström M. Mjörndal T. Dahlqvist R. Nordkvist-Olsson T. (2000). Attitudes to Reporting Adverse Drug Reactions in Northern Sweden. Eur. J. Clin. Pharmacol. 56 (9–10), 729–732. 10.1007/s002280000202 11214784

[B4] Beninger P. Ibara M. A. (2016). Pharmacovigilance and Biomedical Informatics: A Model for Future Development. Clin. Ther. 38 (12), 2514–2525. 10.1016/j.clinthera.2016.11.006 27913029

[B6] Chejor P. (2018). Pharmacovigilance and Adverse Drug Reactions Reporting in Bhutan: A Review of Current Status. Ijopp 11 (2), 67–70. 10.5530/ijopp.11.2.15

[B7] Constant Allabi A. Nwokike J. (2014). A Situational Analysis of Pharmacovigilance System in Republic of Benin. J. Pharmacovigil 02. 10.4172/2329-6887.1000136

[B8] Daley M. F. Narwaney K. J. Shoup J. A. Wagner N. M. Glanz J. M. (2018). Addressing Parents' Vaccine Concerns: A Randomized Trial of a Social Media Intervention. Am. J. Prev. Med. 55 (1), 44–54. 10.1016/j.amepre.2018.04.010 29773490 PMC8606186

[B9] Darrow J. J. Avorn J. Kesselheim A. S. (2020). FDA Approval and Regulation of Pharmaceuticals, 1983-2018. JAMA 323 (2), 164–176. 10.1001/jama.2019.20288 31935033

[B10] Directorate of malaria control Pakistan (2017). Assessment of Therapeutic Efficacy and Safety of Artesunate + Sulfadoxine in Uncomplicated Falciparum Malaria: A Senital Site Based Survey 2017. Available at: http://dmc.gov.pk/documents/pdfs/Drug Efficacy Survey 2017.pdf . (Accessed September 12, 2021).

[B14] Directorate of malaria control Pakistan. (2014). Strategic Plan Malaria Control Program Pakistan: 2015-2020. Available at: http://dmc.gov.pk/documents/pdfs/1National Malaria-Strategic Plan-Pakistan.pdf . (Accessed September 9, 2021).

[B15] DRAP. (2019). Advisory on Registered Medicine. Drug Regul. Auth. Pakistan. Available at: file:///C:/Users/Abbas/Downloads/FORMULATI ONS OF C OFLOX CIN.pdf . (Accessed September 4, 2021).

[B11] DRAP (2021a). List of valid drug manufacturing units operating in Pakistan. Drug Regul. Auth. Pakistan. Available at: https://dra.gov.pk/Home/Licensing#gsc.tab=0 . (Accessed September 2, 2021).

[B16] DRAP (2017b). Minutes for 266th Registration Board Meeting. Drug Regul. Auth. Pakistan. Available at: https://dra.gov.pk/Home/Registration#gsc.tab=0 . (Accessed September 4, 2021).

[B18] DRAP (2016). Minutes of 263rd Meeting of Regsitration Board. Drug Regul. Auth. Pakistan. Available at: https://dra.gov.pk/Home/Registration#gsc.tab=0 . (Accessed September 4, 2021).

[B66] DRAP (2017a). Minutes of 265th meeting of Registration Board. Drug Regul. Auth. Pakistan. Available at: https://dra.gov.pk/Home/Registration#gsc.tab=0 . (Accessed September 4, 2021).

[B12] DRAP (2018). Minutes of the 281st meeting of Registration Board. Drug Regul. Auth. Pakistan. Available at: https://dra.gov.pk/Home/Registration#gsc.tab=0 . (Accessed September 4, 2021).

[B17] DRAP (2021b). Pharmacoviglance Safety Alerts. Drug Regul. Auth. Pakistan. Available at: http://www.dra.gov.pk/Home/PharmacyServices . (Accessed September 1, 2021).

[B13] DRAP (2021c). Provisional Draft List of Registered Drugs. Drug Regul. Auth. Pakistan. Available at: https://dra.gov.pk/Home/Registration#gsc.tab=0 . (Accessed September 2, 2021).

[B19] Glamočlija U. Tubić B. Kondža M. Zolak A. Grubiša N. (2018). Adverse Drug Reaction Reporting and Development of Pharmacovigilance Systems in Bosnia and Herzegovina, Croatia, Serbia, and Montenegro: A Retrospective Pharmacoepidemiological Study. Croat. Med. J. 59 (3), 124–131. 10.3325/cmj.2018.59.124 29972735 PMC6045893

[B22] Hussain R. (2021). Big Data, Medicines Safety and Pharmacovigilance. J. Pharm. Pol. Pract. 14 (1), 48–53. 10.1186/s40545-021-00329-4 PMC8170061 34078480

[B23] Hussain R. Hassali M. A. Hashmi F. Farooqui M. (2018). A Qualitative Exploration of Knowledge, Attitudes and Practices of Hospital Pharmacists towards Adverse Drug Reaction Reporting System in Lahore, Pakistan. J. Pharm. Pol. Pract. 11 (1), 16–10. 10.1186/s40545-018-0143-0 PMC6052559 30034811

[B43] Hussain R. Hassali Mi. Muneswarao J Hashmi F. 2020. Physicians’ Understanding and Practices of Pharmacovigilance: Qualitative Experience from a Lower Middle-Income Country. Int. J. Environ. Res. Public Health 2209. 10.3390/ijerph17072209 PMC7178000 32218355

[B24] Hussain R. Hassali M. A. Hashmi F. Akram T. (2021). Exploring Healthcare Professionals' Knowledge, Attitude, and Practices towards Pharmacovigilance: a Cross-Sectional Survey. J. Pharm. Pol. Pract. 14 (1), 1–13. 10.1186/s40545-020-00287-3 PMC7784002 33397478

[B25] Iffat W. Shakeel S. Naseem S. Imam S. Khan M. (2014). Attitudinal Survey to Assess Medical and Dental Students’ Belief of ADR Reporting in Pakistan. Int. J. Pharm. Pharm. Sci. 6 (5), 279–283.

[B26] Iftikhar S. Sarwar M. R. Saqib A. Sarfraz M. (2018). Causality and Preventability Assessment of Adverse Drug Reactions and Adverse Drug Events of Antibiotics Among Hospitalized Patients: A Multicenter, Cross-Sectional Study in Lahore, Pakistan. PLoS ONE 13, e0199456. 10.1371/journal.pone.0199456 29949616 PMC6021047

[B39] Jha N. Palaian S. Shankar P. R. K. C. S. Kshetry P. B. (2021). Situation analysis of the pharmacovigilance system in Nepal using the indicator-based pharmacovigilance assessment tool (IPAT) J. Pharm. Heal. Serv. Res. 2–3. 10.1093/jphsr/rmab054

[B27] Kabore L. Millet P. Fofana S. Berdai D. Adam C. Haramburu F. (2013). Pharmacovigilance Systems in Developing Countries: An Evaluative Case Study in Burkina Faso. Drug Saf. 36 (5), 349–358. 10.1007/s40264-013-0043-9 23580195

[B53] Khan M. U. Ahmad A. Ejaz A. Ata Rizvi S. Sardar A. Hussain K. (2015). Comparison of the Knowledge, Attitudes, and Perception of Barriers Regarding Adverse Drug Reaction Reporting between Pharmacy and Medical Students in Pakistan. J. Educ. Eval. Health Prof. 12, 28. 10.3352/jeehp.2015.12.28 26072906 PMC4536349

[B28] Lembit R. Santoso B. (2010). Drug Regulation: History, Present and Future. Focus Altern. Complement. Therapies 7 (2), 65–76. 10.1111/j.2042-7166.2002.tb05487.x

[B29] LHC (2012). Punjab Institute of Cardiology (PIC) Defective Drugs Inquiry Report 2011-2012. Lahore High Court. Available at: http://apps.who.int/medicinedocs/documents/s22131en/s22131en.pdf . (Accessed September 15, 2021).

[B30] Mahmood K. T. Amin F. Tahir M. Haq I. U. (2011). Pharmacovigilance - A Need for Best Patient Care in Pakistan. A Review. J. Pharm. Sci. Res. 3, 1566–1584.

[B31] Meher B. (2021). Need of Vibrant Vaccine Pharmacovigilance during Current Global COVID-19 Pandemic: More Than Ever. J. Pharm. Bioall Sci. 13 (1), 1. 10.4103/jpbs.JPBS_416_20 PMC8142916 34084042

[B32] Muhammad K. Saqlain M. Muhammad G. Hamdard A. Naveed M. Butt M. H. (2021). Knowledge, Attitude, and Practices (KAPs) of Community Pharmacists Regarding COVID-19: A Cross-Sectional Survey in 2 Provinces of Pakistan. Disaster Med. Public Health Prep.. 10.1017/dmp.2021.54 PMC8129683 33588970

[B20] Mustafa G. Rasheed S.-U. Aziz M. T. (2013). Adverse Drug Reaction Reporting System At Different Hospitals of Lahore, Pakistan - an Evaluation and Patient Outcome Analysis. J. App Pharm. 5, 9–15. 10.21065/19204159.5.9

[B33] Nisa Z. U. Zafar A. Sher F. (2018). Assessment of Knowledge, Attitude and Practice of Adverse Drug Reaction Reporting Among Healthcare Professionals in Secondary and Tertiary Hospitals in the Capital of Pakistan. Saudi Pharm. J. 26 (4), 453–461. 10.1016/j.jsps.2018.02.014 29844715 PMC5961757

[B34] Nwokike J. Eghan K. (2010). Pharmacovigilance in Ghana: A Systems Analysis. Submitted to the U.S. Agency for International Development by the Strengthening Pharmaceutical Systems (SPS) Program. Arlington, VA: Management Sciences for Health.

[B35] Nwokike J. Joshi M. P. (2010). Pharmacovigilance in Ghana: A Systems Analysis. Submitted to the U.S. Agency for International Development by the Strengthening Pharmaceutical Systems (SPS) Program. Arlington, VA: Management Sciences for Health.

[B36] Olsson S. Pal S. N. Stergachis A. Couper M. (2010). Pharmacovigilance Activities in 55 Low- and Middle-Income Countries: a Questionnaire-Based Analysis. Drug Saf. 33 (8), 689–703. 10.2165/11536390-000000000-00000 20635827

[B37] Opadeyi A. O. Fourrier-Réglat A. Isah A. O. (2018). Assessment of the State of Pharmacovigilance in the South-South Zone of Nigeria Using WHO Pharmacovigilance Indicators. BMC Pharmacol. Toxicol. 19 (1), 27–28. 10.1186/s40360-018-0217-2 29855348 PMC5984375

[B38] Oshikoya K. A. Awobusuyi J. O. (2009). Perceptions of Doctors to Adverse Drug Reaction Reporting in a Teaching Hospital in Lagos, Nigeria. BMC Clin. Pharmacol. 9 (1), 14. 10.1186/1472-6904-9-14 19671176 PMC2731723

[B40] Pitts P. J. (2015). OpenFDA: An Open Question. Ther. Innov. Regul. Sci. 49 (2), 254–255. 10.1177/2168479014555913 30222417

[B41] Provincial Quality Control Unit Punjab (2021). Drug Safety Alerts. Available at: https://sites.google.com/prod/view/pdcup/divisions/drug-safety-alerts . (Accessed September 1, 2021).

[B42] Qato D. M. (2018). Current State of Pharmacovigilance in the Arab and Eastern Mediterranean Region: Results of a 2015 Survey. Int. J. Pharm. Pract. 26 (3), 210–221. 10.1111/ijpp.12372 28737220

[B21] Raza A. Jamal H. (2015). Assessment of Knowledge, Attitudes and Practice Among the Medical and Pharmacy Students towards Pharmacovigilance and Adverse Drug Reactions in Abbottabad, Pakistan. J. Pharmacovigilance 03. 10.4172/2329-6887.1000173

[B45] Rice E. (2007). Dr . Frances Kelsey : Turning the Thalidomide Tragedy into Food and Drug Administration Reform. Int. J. Adv. Pharm. 2 (1), 108–110.

[B46] Shakeel S. Iffat W. Anjum F. Bushra Ra. Ibrahim Sf. Shafiq S. (2014). “Emerging Need of Pharmacovigilance : Perspectives of Future Pharmacist in Pakistan Emerging Need of Pharmacovigilance : Perspectives of Future Pharmacist in Pakistan,” No. June.

[B47] Shamim S. Sharib S. M. Malhi S. M. Muntaha S. U. Raza H. Ata S. (2016). Adverse Drug Reactions (ADRS) Reporting: Awareness and Reasons of Under-Reporting Among Health Care Professionals, a Challenge for Pharmacists. Springer Plus 5 (1). 10.1186/s40064-016-3337-4 PMC5061681 27795920

[B48] Shrestha S. Palaian S. Shrestha B. Santosh K. Khanal S. (2019). The Potential Role of Social Media in Pharmacovigilance in Nepal: Glimpse from a Resource-Limited Setting. Jcdr 13 (3). 10.7860/jcdr/2019/39979.12693

[B49] Statista (2021). Pakistan: Total population from 2016 to 2026. Available at: https://www.statista.com/statistics/383245/total-population-of-pakistan/ . (Accessed September 4, 2021).

[B50] Stergachis A. Bartlein R. J. Dodoo A. Nwokike J. Kachur S. P. (2010). A Situational Analysis of Pharmacovigilance Plans in the Global Fund Malaria and U.S. President's Malaria Initiative Proposals. Malar. J. 9 (1), 148–210. 10.1186/1475-2875-9-148 20509971 PMC2887883

[B57] Strengthening Pharmaceutical Systems (SPS) Program (2009). Indicator-Based Pharmacovigilance Assessment Tool : Manual for Conducting Assessments in Developing Countries. Submitted to the U.S. Agency for International Development by the SPS Program. Arlington, VA: Management Sciences for Health.

[B51] Syed A. Azhar S. Raza M. M. Saeed H. Jamshed S. Q. (2018). Assessment of Knowledge, Attitude and Barriers towards Pharmacovigilance Among Physicians and Pharmacists of Abbottabad, Pakistan. Pharmacy (Basel) 6 (2), 29. 10.3390/pharmacy6020029 PMC6025379 29614725

[B44] The Global Fund (2020). Global Fund Grants in the Islamic Republic of Pakistan: Audit Report. February.

[B5] The Pakistan Business Council (2021). A Health Check for a Better Future: Unleashing the Potential of Pharmaceuticals in Pakistan.

[B52] Tribune T. E. (2012). Tyno syrup: Deaths caused by clotting in heart, reveals post-mortem report. Available at: https://tribune.com.pk/story/472126/nothing-wrong-with-tyno-syrup-forensic-report .

[B54] UMC (2021a). Members of the WHO Programme for International Drug Monitoring. Available at: https://www.who-umc.org/global-pharmacovigilance/members/who-programme-members/ . (Accessed September 21, 2021).

[B55] UMC (2000). Safety Monitoring of Medicinal products: Guidelines for setting up and running a Pharmacovigilance system.

[B56] UMC (2021b). Signal Liberary. Available at: https://www.who-umc.org/research-scientific-development/signal-detection/signal-library/ . (Accessed September 6, 2021).

[B58] WHO (2010). Minimum Requirements for a functional Pharmacovigilance System. World Heal. Organ, 14–15. Available at: https://www.who.int/medicines/areas/quality_safety/safety_efficacy/PV_Minimum_Requirements_2010_2.pdf . (Accessed September 6, 2021).

[B59] WHO (2021a). Pakistan: Health Service Delivery. Available at: http://www.emro.who.int/pak/programmes/service-delivery.html . (Accessed September 12, 2021).

[B60] WHO (2004). Pharmacovigilance : Ensuring the Safe Use of Medicines, 1–6.

[B61] WHO (2002). Pharmacovigilance. “The Importance of Pharmacovigilance - Safety Monitoring of Medicinal Products.” World Health Organization, 1–52. 10.1002/0470853093

[B62] WHO (2021b). Vaccine Safety basics, E-Learnign Course: Rumors and Crises. Available at: https://vaccine-safety-training.org/rumours-and-crises.html . (Accessed August 22, 2021).

[B63] WHO (2006). “The Safety of Medicines in Public Health Programmes: Pharmacovigilance an Essential Tool. WHO Library Cataloguing-in-Publication Data, 61. doi: ISBN 92 4 159391.

[B64] WHO (2015). WHO Pharmacovigilance Indicators: A practical manual for the assessment of Pharmacovigilance system. World Heal. Organ. 300. Available at: https://www.who.int/medicines/areas/quality_safety/safety_efficacy/EMP_PV_Indicators_web_ready_v2.pdf (Accessed September 16, 2021).

[B65] Wilbur K. (2013). Pharmacovigilance in the Middle East: A Survey of 13 Arabic-Speaking Countries. Drug Saf. 36 (1), 25–30. 10.1007/s40264-012-0001-y 23315293

