# Supplementary material for: Assessment of the Current State of Pharmacovigilance System in Pakistan Using Indicator-Based Assessment Tool
Source: Front Pharmacol. 2022 Jan 14;12:789103. doi: 10.3389/fphar.2021.789103 (PMC8795784; doi:10.3389/fphar.2021.789103)
Supplement: Supplementary file 1 [file Table1.docx]

Supplementary Material

| **Study participants for the assessment of current Pharmacovigilance activities in Pakistan** | | |
| --- | --- | --- |
| **A.** | **Pharmacovigilance activities at the national level** | |
|  | **1. Drug Regulatory Authority of Pakistan** | a. Pharmacy Services Division |
|  | **2. National Health Programs.** | 1. National Malaria Control Program (NMCP) 2. National Aids Control Program (NACP) 3. National Tuberculosis Control Program (NTBCP) 4. Expanded Program on Immunization (EPI) 5. Pakistan Polio Eradication Initiative (PPEI) |
| **B.** | **Pharmacovigilance activities at the provincial level** | |
|  | **1. Health Administrative Unit of Pakistan** | 1. Azad Jammu and Kashmir (AJK) 2. Baluchistan, 3. Gilgit Baltistan (GB) 4. Islamabad Capital Territory (ICT) 5. Khyber Pakhtunkhwa (KPK) 6. Punjab 7. Sindh |
|  | **2. Health facilities** | **a. Private hospitals**   1. Agha Khan University Hospital (AKUH), Karachi 2. Quaid-e-Azam international hospital (QIH), Islamabad 3. Shifa International Hospital (SIH), Islamabad 4. Shaukat Khanum Memorial Cancer Hospital (SKMCH), Lahore 5. Rehman Medical Institute (RMI), Peshawar 6. Agha Khan Medical Center (AKMCG) Gilgit, 7. Baluchistan institute of Nephrology and kidney transplant (BINIQ) Quetta, 8. Riaz Hospital (RHM) Mirpur, AJK   **b. Government/Public hospitals**   1. Allied Hospital (AH), Faisalabad 2. Benazir Bhutto Shaheed Hospital (BBH), Rawalpindi 3. District Headquarter Hospital (DHH), Rawalpindi 4. Holy Family Hospital (HFH), Rawalpindi 5. Federal Government Polyclinic Hospital (FGPH), Islamabad 6. Pakistan Institute of Medical Sciences (PIMS), Islamabad 7. Children Hospital (CH), Lahore 8. Jinnah Hospital (JH), Lahore 9. Punjab Institute of Cardiology (PIC), Lahore 10. Jinnah Postgraduate Medical Centre (JPMC), Karachi 11. National Institute of Child Health (NICH), Karachi 12. Hayatabad Medical Complex (HMC), Peshawar 13. DHQ Hospital (DHQHG) Gilgit, 14. Bolan medical complex hospital (BMCH) Quetta 15. DHQ Teaching Hospital (DHQTH) Mirpur AJK |
| **C.** | **Others** | |
|  | **1. Websites of participating organizations** |  |
|  | **2. Official/legal documents** | 1. The Drugs Act 1976 2. The DRAP Act 2012 3. Guidelines of Public Health Programs (PHPs) 4. Pakistan National PV guidelines 5. Punjab PV plans 2017 and 2019 6. Draft PV Rules 2020 7. Fundamentals of PV and its emergence in Punjab 8. National Health vision Pakistan 2016-2025. |
